# Body Weight Misperception and Its Association with Unhealthy Eating Behaviors among Adolescents in China

**DOI:** 10.3390/ijerph15050936

**Published:** 2018-05-08

**Authors:** Hanyi Yan, Yingru Wu, Theresa Oniffrey, Jason Brinkley, Rui Zhang, Xinge Zhang, Yueqiao Wang, Guoxun Chen, Rui Li, Justin B. Moore

**Affiliations:** 1 School of Health Sciences, Wuhan University, Wuhan 430071, China; yanlq7@hotmail.com (H.Y.); yingru.wu@hotmail.com (Y.Wu.); ylcz2920@126.com (X.Z.); elle529@126.com (Y.Wa.); 2 Cerus Consulting, LLC, Winston-Salem, NC 27101, USA; toniffrey@gmail.com; 3 Abt Associates, Durham, NC 27703, USA; Jason_brinkley@abtassoc.com; 4 College of Life Sciences, South-Central University for Nationalities, Wuhan 430074, China; zhangrui@mail.scuec.edu.cn; 5 Department of Nutrition, The University of Tennessee, Knoxville, TN 37996, USA; gchen6@utk.edu; 6 Department of Family & Community Medicine, Wake Forest School of Medicine, Medical Center Boulevard, Winston-Salem, NC 27157, USA; 7 Department of Epidemiology & Prevention, Wake Forest School of Medicine, Winston-Salem, NC 27157, USA; 8 Department of Implementation Science, Wake Forest School of Medicine, Winston-Salem, NC 27157, USA

**Keywords:** body weight misperception, unhealthy eating behaviors, adolescents

## Abstract

This study aims to examine associations between body weight misperception and eating behaviors among Chinese adolescents. Students (*N* = 2641) from a middle school and a high school in Wuhan, China participated in a cross-sectional study in May 2016. A questionnaire based on the World Health Organization’s Global School-Based Student Health Survey was employed to assess responses. Self-reported data, including weight, height, body weight perception, and eating habits, were collected. Body Mass Index (BMI) for age z-score was calculated from self-reported height and weight using WHO AnthroPlus. We used descriptive, logistic regression analysis and a Kappa test to analyze the data using SPSS. Overall, 56.6% of participants did not correctly categorize their weight status; these were much more likely to be girls. Compared with the correctly-perceived group, those who underestimated their weight tended to report eating late at night, having dinners with family, and checking nutrition labels. In contrast, weight overestimating students were less likely to report eating late at night, having breakfasts with family, having dinners with family, and discussing nutrition topics over meals. Body weight misperception was associated with unhealthy eating behaviors among Chinese adolescents.

## 1. Introduction

Adolescence is a critical period of both physical and mental development when lifestyle behaviors are cultivated. As 16.8% of Chinese youth are overweight or obese, and overweight/obese adolescents are more likely to become obese adults [1], it is important to identify factors contributing to weight status in Chinese adolescents. Furthermore, cultural beliefs and beauty ideals are changing, especially in China [2], and many young people are concerned about their body shape and size due to social pressures to conform to a thin ideal body [3,4,5,6,7]. Media representations may adversely affect self-perceptions of weight, thereby exacerbating the weight misperception [8].

Weight misperception is the over or under estimation of one’s weight. The research literature suggests that body dissatisfaction is a common concern for adolescents and young adults from Western countries [9,10], and some reports suggest that adolescents’ weight misperception is increasing [11,12,13,14]. In a recent study conducted in Korea, 49.3% of Korean youth misperceived their weight status, with similar prevalence of over- and under-estimation. It has been indicated that either overestimation or underestimation of body weight is correlated with health issues in adolescents, including depression and certain psychological conditions [15,16,17]. This may in turn affect adolescents’ weight management behaviors [18,19]. In particular, non-overweight adolescents who overestimate their body size may experience body dissatisfaction, leading to greater risk for disordered eating and eating disorders [20]. Conversely, overweight adolescents who underestimate their body size may be less motivated to lose weight, thus increasing chronic disease risk [21].

Like many Western youth, Chinese adolescents and young adults also have expressed dissatisfaction with body size and shape [22,23,24]. Moreover, research has demonstrated that body image concerns are associated with concerns about appearance, low self-esteem, depression, and stress in large samples of Chinese children and adolescents [12,23,25]. In extreme cases, such concerns can lead to eating disorders and even suicidal ideation [11,26]. Significant gender differences in body size misperception have been previously reported in a representative sample of adolescents from Hong Kong [27], with boys less likely to misperceive their body size than girls. Similarly, differences by gender in body size misperception have been reported among 9- to 10-year-old children in Beijing [25].

It is becoming increasingly important to understand the extent of body image misperception and the associations between it and unhealthy eating behaviors in the Chinese adolescent population. However, few previous studies have tested the relationship between weight misperception and eating behaviors in Chinese youth. This study was therefore designed to assess the associations between body weight misperception and unhealthy eating behaviors among a cohort of Chinese adolescents.

## 2. Materials and Methods

### 2.1. Study Population

A cross-sectional survey was conducted at two schools, a middle school and a high school, in Wuhan, Hubei, China, during the late spring/early summer of 2016. The study was conducted in accordance with the Declaration of Helsinki, and the protocol was approved by the Ethics Committee of Wuhan University (ethical approval code: 2016031269) and medical school district administrators. All adolescents (*N* = 3059) enrolled in grades 7–12 were invited to participate in the study via a recruitment letter and consent form sent home to parents; written informed consent was obtained from a parent or guardian. For those providing consent, a survey was sent home to be completed and returned to school. Of those who consented, 149 respondents did not return the questionnaire, and 258 respondents did not answer questions related to key independent or dependent variables (e.g., weight, grade, sex, eating behaviors, physical activity). Ten respondents older than 19 years of age and one respondent younger than 12 were excluded; this resulted in a final sample size of 2641 youth aged 13–18 years (91.5% of those eligible).

### 2.2. Measures

The school and grade of each student were recorded by study staff; date of birth, sex, height, and weight were self-reported. Age, body mass index (BMI: weight in kg/height in m^2^), and sex- and age-standardized BMI (BMI z-score, or BAZ) were calculated [28]. Weight status was divided into three categories: underweight (BAZ ≤ −2), normal weight (−2 < BAZ < 1), and overweight (BAZ ≥ 1).

The questions, answer options, and the categories of information collected are shown in the Appendix A. The questions about behaviors were grouped by the answer options but not relevant to its negative or positive association.

#### Misperception of Body Weight

The outcome of self-perceived weight status was classified as: underestimation, correct estimation, and overestimation. For example, normal-weight participants were classified in the overestimation category if their self-evaluated weight status was overweight/obese. Overweight and obese participants were placed in the underestimation category if they reported themselves as being normal weight or underweight.

### 2.3. Statistical Analysis

The Kappa statistic was used to account the consistency between objective and self-perceived weight status. Binary logistic regression was used to analyze the associations between misperception of body weight and eating behaviors, and attitudes of losing or gaining weight. Multiple logistic regression was used to analyze the associations between misperception of body weight and the frequency of every kind of food. Separate analyses were conducted for males and female participants. Models were adjusted for age, sex, physical activity, and whether the person reported eating less food or lower-fat food to lose weight. Distributions and frequencies for each category of variables were examined. Kappa, odds ratios (OR), and *p*-values were calculated where appropriate to assess the relationships between weight misperception and the dependent variables. All analyses were conducted using a combination of SPSS v22.0 (IBM Corporation, Armonk, NY, USA) [29] and JMP v13.0 (SAS Institute Inc., Cary, NC, USA) [30].

## 3. Results

### 3.1. Characteristics of the Study Population

Characteristics of the study population are summarized in Table 1. Based on BAZ, about 80% of respondents were of normal weight status. However, based on self-evaluation, about 23% of participants consider themselves underweight and 45% considered themselves either overweight or obese.

### 3.2. Proportions of Weight Status Misperception 

Table 2 is a cross-tabulation of self-reported weight status and self-perceived weight status. The agreement between objective weight status and self-perceived weight status was very low. The descriptive statistics and proportions of each weight status misperception pattern are shown in Table 3. Nearly half of female participants overestimated their weight. Thin and overweight or obese adolescents were more likely to correctly perceive their weight. On the other hand, participants of normal weight are more likely to incorrectly estimate their weight, especially overestimate.

### 3.3. Associations between Attitudes about Losing Weight and Weight Misperception among Normal-Weight Participants

Associations between attitudes of losing weight and weight misperception among normal weight participants are presented in Table 4. Normal-weight participants who perceived themselves as overweight or obese were significantly more likely to eat less food or lower-fat food to lose weight compared to those who underestimated or correctly estimated their weight; these were more likely to be male respondents. Normal-weight participants who perceived themselves as thin were significantly less likely to eat less food or lower-fat food to lose weight, especially female participants.

### 3.4. Associations between Eating Behaviors and Weight Misperception

Associations between eating behaviors and weight misperceptions are shown in Table 5. Compared with the correctly-perceived group, weight-overestimating respondents were less likely to report usually having food late at night, having breakfast or dinner with family, and discussing nutrition topics over meals; they also were more likely to have takeout food. Compared with the correctly-perceived group, weight-underestimating students were more likely to eat food late at night, have dinners with family, check nutrition labels, and discuss nutrition topics over meals.

### 3.5. Associations between Eating Behaviors and Weight Status Misperception by Sex

Among males, compared with the correctly-perceived group, weight-overestimating respondents were less likely to have breakfast every day and have dinners with family. Weight-underestimating male respondents were more likely to report eating late at night, checking nutrition labels, discussing nutrition topics over meals, and taking nutritional supplements (Table 6). Among females, compared with the correctly-perceived group, weight-overestimating respondents were less likely to eat food late at night, discuss nutrition topics over meals, and more likely to have takeout food. Weight-underestimating female respondents were more likely to consume food late at night, have dinners with family, and discuss nutrition topics over meals (Table 6).

## 4. Discussion

Nearly 57% of our respondents showed weight misperception, and consistency between objective weight and self-perceived weight status was very low in this group. A considerable number of normal-weight individuals erroneously reported their weight status (22.7% underestimated and 41.0% overestimated). Several studies have suggested that sociocultural factors may explain body dissatisfaction among young men and women in China. For example, Chen et al. [23] found that teasing and social pressure to be thin directly predicted body dissatisfaction in a large sample of Chinese adolescents and young adults. Jackson et al. found that media pressure about appearance predicted body dissatisfaction among Chinese female and male college students [22]. In that study, over one-third of participants overestimated their weight status. What’s more, students who overestimated their status in senior high school were more likely to overestimate their weight as they grew older. These trends may be due to changing cultural beliefs and beauty ideals, as many young people are concerned about their body shape and size due to social pressures to conform to a thin body ideal [3,4,5,6,7]. There is evidence that pressure about appearance from the media, friends, and family deliver unrealistic societal standards of physical beauty [2].

In our study, a quarter of overweight or obese participants underestimated their weight. This is troubling, as correct perception of one’s weight is associated with maintenance of a healthy lifestyle. As reported by Skinner et al. [31] and Jones and colleagues [32], individuals who misperceived their weight report fewer weight concerns, less control over emotional distress, and related overeating. In a separate study, overweight youngsters who perceived their weight accurately consumed fewer calories, reported more physical activity, and recorded more weight loss [33]. In the current study, overweight or obese participants were also more likely to report indulging in unhealthy food choices such as fast food and/or sugar sweetened beverages, snacking at inappropriate times, and engaging in more sedentary behaviors. This has important clinical ramifications, as the current findings provide several behaviors that could be targeted through behavioral counselling by clinicians or dieticians, such as having food late at night, having dinners with families, and having takeout food.

In previous studies, rates of weight misperception ranged from 46.0% among US adolescents [34], 40.0% in young Dutch adolescents [35], and approximately 45.0% (47.4% of boys and 44.2% of girls) in 6 different central-eastern European countries (Hungary, Slovakia, Czech Republic, Romania, Ukraine, and Poland) [36]. For example, a study of African-American adolescents in the U.S. [37] showed that 67.2% correctly judged their weight status, 27.2% underestimated it, and only 5.6% overestimated their weight status [38]. The difference between findings in this study and others might be due to ethnic differences or the higher prevalence of overweight adolescents, which was 39.8% in the study by Wang et al. [37] versus 14.5% in the present study. However, in another study among normal-weight adolescents in the U.S., only 16.2% overestimated their weight [20], much lower than the 32.3% we report here.

In this study, adolescents who overestimated their weight were more likely to have takeout food, not have breakfast and dinner with family, not discuss nutrition topics over meals, and not have snacks at night; those who underestimated their weight had the opposite results. Among those who overestimated their weight, male respondents were less likely to have breakfast every day and have dinners with family, while female respondents were less likely to eat food late at night or discuss nutrition topics over meals, and more likely to have takeout food. Among weight-underestimating respondents, participants were more likely to discuss nutrition topics over meals and have food late at night regardless of gender. However, female participants were more likely to have dinners with family, while male participants were more likely to take nutritional supplements and check nutrition labels. Research regarding eating behaviors according to weight-perception status is limited. A few related studies have been reported for Chinese [14] and African-American [38] adolescents. For example, it was found that unhealthy eating habits, such as drinking less milk and consuming less fruit, were prevalent in weight-underestimating African-American girls. However, African-American boys who underestimated their weight were less likely to eat snacks [38]. In a previous study based on US nationally representative data, accurate weight perception was associated with healthy weight-related behaviors, including more dietary intake of fruit and vegetables and more physical activity, in both boys and girls [33]. Therefore, the formation of healthy dietary habits can be at least partially attributed to an accurate body weight perception.

The two important findings in our study were the high prevalence of overestimation amongst female respondents and underestimation among male respondents. This was in accordance with previous studies [13,14,33,36]. Among normal-weight participants, both those who underestimated and overestimated their weight reported trying to lose weight through eating less food or eating lower-fat food, especially girls. The differences observed by gender may reflect social norms that accept a larger range of weight statuses in boys while simultaneously enforcing a ‘ideal thin’ physique for girls. This might be affected by mass media and reflect a growing social issue in China. Also, exposure to media from either USA or Asian countries/regions (Japan, Korea, Taiwan, and Hong Kong) leaded to perceive or misperceive overweight in girls and underweight in boys [14]. The ideal body image has been for men to be muscular and women to be thin with the rise and influence of mass media throughout past decades. Consistently, we found that weight underestimating males seemed to be more likely to exercise to develop muscles, whereas weight overestimating females were more likely to be involved in vigorous physical activity to lose weight.

To help adolescents to keep healthy both mentally and physically, the following aspects should be considered. Firstly, general education about weight perception is essential. A correct self-perception of weight status is a necessary prerequisite to good health, as suggested by Cai et al. [39] and Fan et al. [40]. Moreover, the government should require schools to provide abundant healthy food with the introduction of healthy food options in the school café to make sure students eat well. Furthermore, appropriate education about physical activity is also needed, so that students do not hurt themselves. Finally, further large-scale prospective studies are needed for the government to assess adolescents’ dietary structure. For parents, it is important to promote a healthy family lifestyle.

There are several limitations in this study. First, the cross-sectional design of our study limits the ability to determine causal relationships. Most of these data were self-reported, and therefore the validity of the BMI categorization is unknown. Moreover, the sample is limited to Wuhan and may not be representative of all Chinese adolescents. Finally, the self-designed questionnaire may raise potential issues related to reliability and validity. A cohort study using a previously validated questionnaire could improve the quality of data.

## 5. Conclusions

In this cohort of Chinese middle- and high-school students, more than half did not correctly perceive their own body weight. Those who were inaccurate about their weight were much more likely to be girls. Both underestimation and overestimation could result in inappropriate weight-control behaviors and unhealthy eating behaviors. Thus, programs and comprehensive interventions aiming to correct adolescents’ weight misperception are important for their healthy growth and development.

## Figures and Tables

**Table 1:** Characteristics of the study population (*N* = 2641).

Characteristics	Whole Sample (*N* = 2641)		Male (*n* = 1399)		Female (*n* = 1242)	
	*n*	%	*n*	%	*n*	%
Gender						
Male	1399	53.0	-	-	-	-
Female	1242	47.0	-	-	-	-
Grade						
Middle school	1121	42.4	615	44.0	506	40.7
High school	1520	57.6	784	56.0	736	59.3
Sleeping time						
Short (<8 h/day)	2143	81.1	1082	77.8	1054	84.9
Long (≥8 h/day)	498	18.9	310	22.2	188	15.1
Physical Activity						
Active	1025	38.8	640	45.7	385	31.0
Not Active	1616	61.2	759	54.3	857	69.0
Objective weight status (BAZ)						
Underweight (≤−2)	103	3.9	64	4.6	39	3.1
Normal (−2~1)	2156	81.6	1051	75.1	1105	89.0
Overweight/obese (≥1)	382	14.5	284	20.3	98	7.9
Self-perceived weight status						
Underweight	604	22.9	422	30.2	182	14.7
Normal	854	32.3	469	33.5	394	31.0
Overweight/obese	1183	44.8	508	36.3	675	54.3
Having food late at night						
Usually (≥2 times/week)	1190	45.1	725	51.8	465	37.4
Rarely (<2 times/week)	1451	54.9	674	48.2	777	62.6
Having taken-out food						
Usually (≥6 times/month)	1174	44.5	606	43.3	568	45.7
Rarely (<6 times/month)	1467	55.5	793	56.7	674	54.3
Having breakfast						
Usually (>5 times/week)	2018	76.4	1081	77.3	937	75.4
Rarely (≤5 times/week)	623	23.6	318	22.7	305	24.6
Having breakfast with family						
Usually (≥2 times/week)	1128	42.7	641	45.8	487	39.2
Rarely (<2 times/week)	1513	57.3	758	54.2	755	60.8
Having dinner with family						
Usually (≥2 times/week)	1539	58.3	892	63.8	647	52.1
Rarely (<2 times/week)	1102	41.7	507	36.2	595	47.9
Checking the nutrition labels						
Usually	885	33.5	474	33.9	411	33.1
Rarely	1756	66.5	925	66.1	831	66.9
Discussing nutrition topics over meals						
Usually	531	20.1	295	21.1	236	19.0
Rarely	2110	79.9	1104	78.9	1006	81.0
Watching TV or videos on their phone over meals						
Usually	525	19.9	296	21.2	229	18.4
Rarely	2116	80.1	1103	78.8	1013	81.6
Taking nutritional supplements						
Yes	962	36.4	510	36.5	452	36.4
No	1679	63.6	889	63.5	790	63.6
Eating less food or lower-fat food to lose weight						
Yes	767	29.0	342	24.4	425	34.2
No	1874	71.0	1057	75.6	817	65.8

Note: Data were collected from a middle school and a high school, in Wuhan, China, during the late spring/early summer of 2016.

**Table 2:** Relationships between objective and self-perceived weight status (*N* = 2641).

Objective Weight Status	Self-Perceived Weight Status			Total
	Thin	Normal	Overweight/Obesity	
Thin	71	25	7	103
Normal	489	783	884	2156
Overweight/obesity	44	46	292	382
Total	604	854	1183	2641

Note: Kappa = 0.145, *p* < 0.001. Data were collected from a middle school and a high school, in Wuhan, China, during the late spring/early summer of 2016.

**Table 3:** Proportions of weight status misperception (*N* = 2641).

Characteristics	Underestimate		Correctly Estimate		Overestimate	
	*n*	%	*n*	%	*n*	%
All (*N* = 2641)	579	21.9	1146	43.4	916	34.7
Gender						
Male	410	29.3	680	48.6	309	22.1
Female	169	13.6	466	37.5	607	48.9
Grade						
Middle school	262	23.4	523	46.6	336	30.0
Grade 7	81	23.5	158	45.8	106	30.7
Grade 8	94	25.7	169	46.2	103	28.1
Grade 9	87	21.2	196	47.8	127	31.0
Senior high school	317	20.9	623	41.0	580	38.1
Grade 10	70	18.6	167	44.4	139	37.0
Grade 11	152	22.6	265	39.4	256	38.0
Grade 12	95	20.2	191	40.6	185	39.2
BAZ						
≤−2	0	0.0	71	68.9	32	31.1
−2~1	489	22.7	783	36.3	884	41.0
≥1	90	23.6	292	76.4	0	0.0

Notes: Data were collected from a middle school and a high school, in Wuhan, China, during the late spring/early summer of 2016. BAZ: BMI z-score.

**Table 4:** Eating less food or lower-fat food lower to lose weight: normal-weight participants (Logistic regressions, *N* = 2641).

	Eating Less Food or Lower-Fat Food to Lose Weight					
	All		Male		Female	
	OR (95% CI)	*p*-Value	OR (95% CI)	*p*-Value	OR (95% CI)	*p*-Value
Correctly estimate	1 [Ref]		1 [Ref]		1 [Ref]	
Underestimate	0.57 (0.42–0.78)	0.001 **	0.71 (0.46–1.10)	0.126	0.50 (0.31–0.80)	0.004 **
Overestimate	2.27 (1.82–2.83)	<0.001 **	3.71 (2.58–5.34)	<0.001 **	1.67 (1.27–2.21)	<0.001 **

Notes: Adjusted for gender, grade, and physical activity. Correctly perceived weight was used as the control group. Boldface indicates statistical significance (** *p* < 0.01). Data were collected from a middle school and a high school, in Wuhan, China, during the late spring/early summer of 2016.

**Table 5:** Eating behaviors associated with weight misperception among participants (Logistic regressions, *N* = 2641).

	Eating Behaviors					
	Usually having food late at night		Usually having takeout food		Having breakfast everyday	
	OR (95% CI)	*p*-Value	OR (95% CI)	*p*-Value	OR (95% CI)	*p*-Value
Correctly estimate	1 [Ref]		1 [Ref]		1 [Ref]	
Underestimate	1.32 (1.07–1.62)	0.008 **	0.93 (0.76–1.14)	0.479	0.80 (0.63–1.02)	0.066
Overestimate	0.78 (0.65–0.94)	0.009 **	1.28 (1.07–1.54)	0.007 **	0.88 (0.71–1.09)	0.255
	**Usually having breakfasts with family**		**Usually having dinners with family**		**Usually checking nutrition labels**	
	**OR (95% CI)**	***p*-Value**	**OR (95% CI)**	***p*-Value**	**OR (95% CI)**	***p*-Value**
Correctly estimate	1 [Ref]		1 [Ref]		1 [Ref]	
Underestimate	1.17 (0.94–1.45)	0.161	1.49 (1.18–1.88)	0.001 **	1.34 (1.08–1.65)	0.007 **
Overestimate	0.78 (0.64–0.95)	0.013 *	0.73 (0.60–0.89)	0.002 **	0.88 (0.72–1.07)	0.198
	**Usually discussing nutrition topics over meal**				**Usually watching TV or phones over meal**	
	**OR (95% CI)**		***p*-Value**		**OR (95% CI)**	***p*-Value**
Correctly estimate	1 [Ref]				1 [Ref]	
Underestimate	1.55 (1.22–1.97)		<0.001 **		0.98 (0.77–1.27)	0.898
Overestimate	0.73 (0.58–0.92)		0.009 **		1.00 (0.79–1.25)	0.968

Notes: Adjusted for gender, grade, physical activity, and whether eating less food or lower-fat foods to lose weight. Correctly perceived weight was used as the control group. Boldface indicates statistical significance (* *p* < 0.05; ** *p* < 0.01). Data were collected from a middle school and a high school, in Wuhan, China, during the late spring/early summer of 2016.

**Table 6:** Association between eating behaviors and weight status misperception among male and female respondents (Logistic regressions, *N* = 2641).

Characteristics	Male				Female			
	Underestimated Weight		Overestimated Weight		Underestimated Weight		Overestimated Weight	
	OR (95% CI)	*p*-Value	OR (95% CI)	*p*-Value	OR (95% CI)	*p*-Value	OR (95% CI)	*p*-Value
Usually having food late at night	1.29 (1.00–1.66)	0.050 *	0.82 (0.62–0.19)	0.169	1.45 (1.02–2.08)	0.041 *	0.76 (0.59–0.98)	0.034 *
Usually having takeout food	0.87 (0.68–1.12)	0.291	1.20 (0.91–1.57)	0.202	0.996 (0.70–1.42)	0.981	1.36 (1.06–1.73)	0.015 *
Having breakfast everyday	0.84 (0.63–1.14)	0.267	0.69 (0.51–0.95)	0.023 *	0.68 (0.45–1.01)	0.054	1.04 (0.78–1.40)	0.795
Usually having breakfasts with family	1.14 (0.88–1.49)	0.315	0.75 (0.56–1.01)	0.056	1.21 (0.83–1.76)	0.329	0.81 (0.62–1.06)	0.117
Usually having dinners with family	1.25 (0.94–1.66)	0.127	0.70 (0.52–0.95)	0.020 *	2.12 (1.40–3.19)	<0.001 **	0.79 (0.60–1.03)	0.084
Usually checking nutrition labels	1.40 (1.08–1.81)	0.011 *	0.93 (0.69–1.24)	0.609	1.20 (0.82–1.74)	0.350	0.83 (0.64–1.08)	0.169
Usually discussing nutrition topics over meal	1.50 (1.12–2.02)	0.007 **	0.75 (0.52–1.08)	0.120	1.67 (1.10–2.51)	0.015 *	0.72 (0.52–0.995)	0.047 *
Usually watching TV or phones over meal	1.04 (0.77–1.41)	0.783	1.08 (0.78–1.51)	0.643	1.42 (0.97–2.08)	0.075	1.19 (0.92–1.54)	0.178
Usually taking nutritional supplements	1.50 (1.16–1.94)	0.002 **	1.12 (0.84–1.50)	0.425	0.88 (0.56–1.38)	0.575	0.92 (0.67–1.26)	0.583

Notes: Correctly perceived weight was used as the control group. Boldface indicates statistical significance (* *p* < 0.05; ** *p* < 0.01). Data were collected from a middle school and a high school, in Wuhan, China, during the late spring/early summer of 2016.

## Appendix A

Body Weight Misperception Questionnaire for Adolescents in Wuhan, China.

**Table:**

Questions	Answer Options	Answer Categories
**Self-Perceived Weight Status**		
How do you describe your weight?	very underweight;	underweight (slightly underweight or very underweight); normal (about right weight); overweight/obese (slightly overweight or very overweight) [41]
	slightly underweight;	
	about the right weight;	
	slightly overweight;	
	very overweight [41,42]	
**Eating Behaviors**		
(1) How many times a month do you eat takeaway food?	no more than 1 time/month;	Usually; Rarely
	2~5 times/month;	
	6~10 times/month;	
	11~15 times/month;	
	almost every day	
(2) How many days did you buy snacks from supermarkets, street vendors, or restaurants during the past week?	0, 1 day;	
	2 days;	
	3 days;	
	4 days;	
	no less than 5 days	
(3) How many times did you have (a) breakfast; (b) food late at night; (c) breakfast with parents; (d) dinner with parents in a week?	once a week;	
	2~3 times a week;	
	4~5 times a week and every day	
(4) Did you (a) look at the nutritional information on food packaging; (b) discuss the topic of diet nutrition at mealtime; (c) watch a TV/phone/computer over a meal; (d) take nutritional supplements?	never;	
	once in a while;	
	usually and always	
**Attitudes to Losing or Gaining Weight**		
During the past 30 days, did you take measures (eat less/more food, food with fewer/more calories, foods low/high in fat or take any pills, powders, or liquids without a doctor’s advice) to lose/gain weight?	yes;	yes;
	no	no
Physical activity: Since strenuous PA is strongly [43] and independently associated with markers of cardiometabolic health [44], and can be more reliably assessed than light or moderate PA [45], we assessed only strenuous PA [45]. Strenuous activity was defined as sports, games, or dance that made respondents breathe hard, make their legs feel tired, or made them sweat [46].		
Are you usually engaged in strenuous activity, equal to or more than three days a week?	yes;	yes;
	no	no
